# Vaccine Hesitancy at Nine Community Sites Across the United States, Early in COVID-19 Vaccine Rollout

**DOI:** 10.1007/s40615-024-02172-0

**Published:** 2024-09-12

**Authors:** Aneeka Ratnayake, Julie H. Hernandez, Jessica Justman, Jason E. Farley, Yael Hirsch-Moverman, Ken Ho, Stockton Mayer, Abiodun Oluyomi, Magdalena E. Sobieszczyk, Shobha Swaminathan, Timothy Skalland, Jean de Dieu Tapsoba, the CoVPN 5002 Study Team, Patricia J. Kissinger

**Affiliations:** 1 School of Public Health and Tropical Medicine, Tulane University, 1440 Canal Street, Suite 2004, New Orleans, LA 70112 USA; 2 ICAP at Columbia University, Mailman School of Public Health, New York, USA; 3 Division of Infectious Diseases, Columbia University Vagelos College of Physicians & Surgeons, Columbia University Irving Medical Center, Aaron Diamond AIDS Research Center, New York, USA; 4 Center for Infectious Disease and Nursing Innovation, Johns Hopkins School of Nursing, Baltimore, USA; 5 Department of Epidemiology, Columbia University Mailman School of Public Health, New York, USA; 6 Department of Medicine, University of Pittsburgh, Pittsburgh, USA; 7 University of Illinois College of Medicine, Chicago, USA; 8 Baylor College of Medicine, Houston, USA; 9 Rutgers New Jersey Medical School, Newark, USA; 10 Fred Hutchinson Cancer Center, Seattle, USA

**Keywords:** COVID-19, Vaccine, Hesitancy, US, Time-location sampling, Community survey

## Abstract

**Background:**

Vaccine hesitancy has been a significant concern throughout the COVID-19 pandemic. Vaccine hesitancy can be attributed to lack of confidence in vaccines, complacency about the health threat, or lack of convenience of vaccination. To date, few studies have used methods designed to include populations underrepresented in research when identifying factors associated with vaccine hesitancy.

**Methods:**

Between January and July 2021, potential participants were recruited from community venues selected through time-location sampling in 15 defined communities in the United States. Study staff administered a questionnaire on demographics, COVID-19 behaviors and attitudes, and vaccination status or intention to consenting individuals. Vaccine hesitancy was analyzed among those age 18 years and older from nine of the 15 sites and was defined as self-reported neutral, unlikely, or very unlikely vaccine intention. Logistic regression modeling, adjusted for site, identified factors associated with vaccine hesitancy.

**Results:**

Among 11,559 individuals, vaccine hesitancy by site ranged from 8.7 to 31.1%. Vaccine hesitancy was associated with being Black compared to White, being White compared to Asian, younger age, unstable housing, being unemployed, lower income, having a disability, providing care in home, not reporting inability to visit sick or elderly relatives during the pandemic, not reporting increased anxiety during the pandemic, and not spending more time with loved ones during the pandemic.

**Conclusions:**

In these selected US communities, early in vaccine rollout, there were significant racial disparities in vaccine hesitancy. Additionally, individuals who were more marginalized due to their socioeconomic status were more likely to report vaccine hesitancy. Vaccine campaigns should make efforts to remove barriers to vaccination, by improving convenience.

**Supplementary Information:**

The online version contains supplementary material available at 10.1007/s40615-024-02172-0.

## Background

COVID-19 vaccine hesitancy emerged as a significant concern during the pandemic as it resulted in sub-optimal vaccine uptake and, consequently, increased transmission of infection, higher rates of hospitalization and death, and the emergence of new variants [1]. The WHO Strategic Advisory Group of Experts on Immunization group has proposed the model of complacency, confidence, and convenience to understand vaccine hesitancy generally [2].

Complacency refers to the lack of perceived importance of vaccination, where individuals do not feel that there is a particular risk to themselves if they remain unvaccinated. For those who are complacent, vaccination may also not be seen as a necessary preventative method and other health priorities may be seen as more important. For example, those who previously tested positive for COVID-19 were more likely to express vaccine complacency than those who had never tested positive [3]. Vaccine complacency varies by age, likely because adverse health outcomes of most vaccine-preventable illness are typically less frequent among younger adults [4]. Complacency can be offset by appealing to individuals’ desires to protect their families or communities or by offering them incentives to get vaccinated [5–7].

Confidence refers to the trust individuals have in the health system and the vaccines that are being offered to them. Historic medical mistrust has been a key driver of lower vaccination rates among Black, Indigenous, and Hispanic individuals in the United States (US), as there have been unethical experiments conducted among these groups in the past [8, 9]. Specifically, confidence in COVID-19 and other vaccines has been shown to be lower among Black individuals than their White counterparts [10–12]. These racial disparities were a significant concern in the US during the COVID-19 pandemic, given the disproportionate impact of this disease on communities of color [13, 14]. Additionally, misinformation (unintentionally sharing incorrect information) and disinformation (intentionally sharing false information) undermine confidence in vaccines, as individuals are told that the vaccines are unsafe or statistics are grossly distorted to misrepresent vaccines’ safety and/or efficacy [15, 16]. Disinformation is profitable, where individuals promote disinformation and sell vaccine alternatives instead [17, 18]. During both the Delta and the initial Omicron variant waves, there was a significant increase in the number of groups spreading misinformation and disinformation on online platforms like Facebook [19, 20]. Increased COVID-19 vaccine hesitancy may have been due to decreased confidence because of misinformation and disinformation and the politicization of the pandemic, where political conservatism emerged as a predictor of lack of vaccine confidence during the COVID-19 pandemic [21–24]. Approaches to combat lack of confidence include community engagement and peer education through trusted stakeholders, whereby vaccinated individuals explain their decision to those expressing lack of confidence; incorporating consideration of anxieties and emotions in discussions of vaccine hesitancy; and transparently labeling mis- and disinformation in online sources [25–28].

The final pillar of this model, convenience, refers to barriers and facilitators to accessing a vaccine. This could include the cost of transportation, the need for time off from work, the need for computer literacy to book an appointment, and the challenge of competing interests (like childcare, lack of paid sick time, and lack of insurance) [29]. It is likely that issues of convenience disproportionately affected marginalized communities, including communities of color, due to social determinants of health [30]. Combating issues around convenience would require structural approaches, like ride-share rebates for vaccine appointments, multiple options for vaccine appointment booking, community vaccine pop-up sites, and childcare options [8, 28].

Though many studies have examined COVID-19 vaccine hesitancy, most have not done so among the general population, including populations underrepresented in research, such as people of color, those with lower income, or those facing unstable housing; rather, existing studies have focused predominantly on more affluent and predominantly White communities [31, 32]. We analyzed community data from the COMPASS study [33], a SARS-CoV-2 seroprevalence survey that used time-location sampling of the general population in 15 US communities, to compare correlates of vaccine hesitancy among marginalized populations with correlates found in studies using other sampling strategies.

## Methods

### Site Selection

**Fig. 1:**
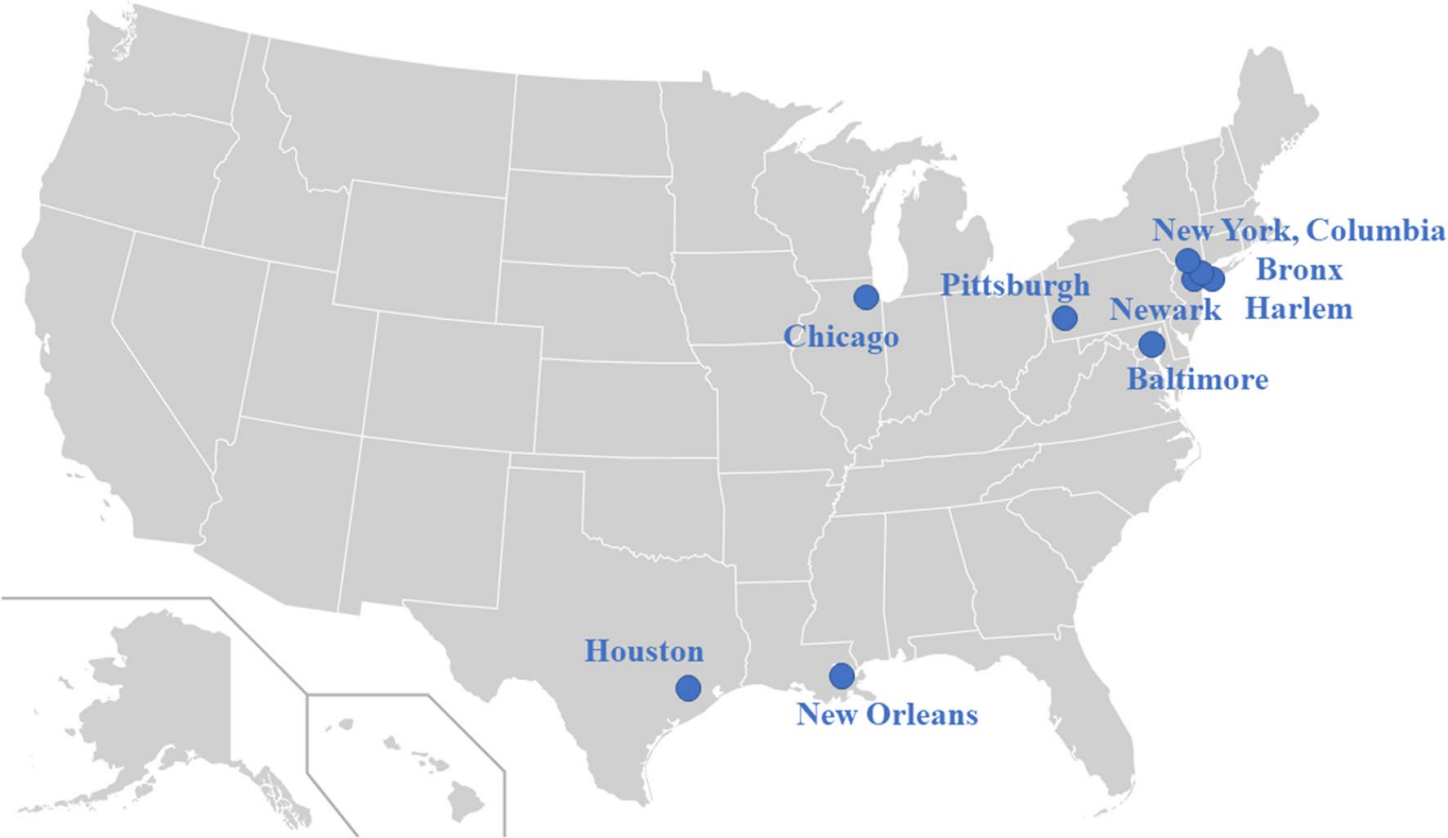
COMPASS sites included in the present study

This study used data from the Community Prevalence of SARS-CoV-2 study (COMPASS) to assess factors associated with early or planned vaccine uptake compared to planned vaccine refusal or neutrality. Study methods are detailed elsewhere [33], but in summary, COMPASS was a multisite, cross-sectional study that aimed to estimate the prevalence of SARS-CoV-2 infection antibodies in 15 large, mostly urban communities surrounding clinical research sites in the United States (Fig. 1). Data collection for COMPASS occurred from January through July 2021, and the study used time-location sampling to recruit participants from the general population, including populations often underrepresented in research. All 15 sites that took part in COMPASS were invited to join the present study and nine accepted. The present study therefore utilized data from Baltimore, MD, Chicago, IL, Houston, TX, New Orleans, LA, Newark, NJ, Pittsburgh, PA, and three sites in New York City, NY: the Bronx, Harlem, and NY-Columbia COMPASS sites.

In the formative phase of COMPASS, the catchment area for each site was defined as the postal (ZIP) code of the clinical research site plus all contiguous ZIP codes, encompassing a population of ~ 150,000. Community venues were defined as public or semi-public spaces (e.g., parking lots of churches or commercial venues) that were safe and accessible to the general public with sufficient open space to allow for the survey and testing operations. Additionally, time blocks during which these venues would be open and accessible were determined. Final validation for the sites included obtaining permission from the building owner or public space supervisor. Community venues were randomized on a weekly basis, with an ordered list generated for each day of planned study activities and for each time block during which a venue would be frequented. During each time block, venues were attempted in order that they appeared on the randomized list, and if it was not possible to use a venue for data collection, the subsequent one would be attempted.

### Data Collection Procedures

Upon arrival at a community venue, the research team attempted to recruit all adults (18 years or older) and all children (2 months or older). The total number of individuals who were solicited at each venue was recorded. If an individual agreed to participate, they were screened to determine eligibility and, if eligible, underwent an informed consent process [34, 35]. Once consent was obtained, individuals completed an interviewer-administered survey that elicited information on demographics, medical status, COVID-19 beliefs and behaviors, and vaccine status or intent. For the present analysis, only survey information from adults 18 years or older was analyzed, as minors required parental consent to receive the COVID-19 vaccines and thus were not in control of their vaccination decisions.

### Outcomes and Data Analysis

At the time of data collection, in the first half of 2021, COVID-19 vaccines were not uniformly available to all. The main outcome of this analysis, vaccine hesitancy, was measured through a survey question asking, “On a scale of 1 to 5, 1 being very likely, 3 being neutral, and 5 being very likely, how likely are you to get an approved COVID-19 vaccine in the future?” At the end of March 2021, the questionnaire was updated to allow participants to self-report whether they had received an “approved” vaccine, i.e., a Food and Drug Administration-authorized COVID-19 vaccine. Vaccine hesitancy was categorized as the binary outcome of “hesitant” or “non-hesitant.” Individuals were considered hesitant if they reported being “neutral,” “unlikely,” or “very unlikely” to receive a COVID-19 vaccine. Individuals were considered non-hesitant if they reported having received a COVID-19 vaccine or were “likely” or “very likely” to do so.

Independent variables were selected based on the model of confidence, complacency, and convenience [2]. Variables were selected through careful consideration of each variable available in the survey in consultation with existing literature, with some variables being attributable to multiple pillars of the confidence, complacency, and convenience model (Table 1). Covariates included age, race, socioeconomic status, medical conditions, and COVID-19-related behaviors. Race was categorized into non-Hispanic White, Hispanic White, Hispanic or non-Hispanic Black, Asian, or Other/Mixed (which included those who did not report their race). Unstable housing was defined as reporting living in a hotel or motel, a drug recovery house, a transitional house, or in a homeless shelter. Individuals were asked (Yes/No) if they needed to care for someone in their home due to COVID-19. Essential worker status was determined through self-report, where participants who reported employment were asked (Yes/No) if they considered themselves an essential worker. A high-risk medical condition was defined as reporting any of the following: asthma, other chronic lung disease, heart disease, hypertension (high blood pressure), cancer chemotherapy in the last 12 months, immunosuppression condition, HIV, diabetes, kidney or renal disease, liver disease, sickle cell disease, obesity, mental health condition, substance use disorder, other chronic medical condition. Time spent in crowds and attending religious services in the 7 days prior to enrollment was each measured on a five-point scale, where response options included 1—never, 2—almost never, 3—some of the time, 4—most of the time, and 5—all of the time.

**Table 1:** Rationale for variable selection, based on the model of confidence, complacency and convenience and existing literature

Variable assessed in COMPASS	Model pillar	Rationale for categorization of variable
Cared for someone in their home (of any age)	Convenience	Individuals responsible for caring for a family member at home may not have been able to take time off to receive their vaccine or may have been concerned about potential side effects [36]
Employment	Convenience	Vaccines may have been less accessible to those with unstable employment (e.g., if they were unable to take time off or did not have adequate income to support transportation costs) [37, 38]
Housing situation	Convenience	Individuals without stable housing may not have had adequate means to get the vaccine, and recover conveniently, and have been shown to have difficulty prioritizing health needs [39]
Income	Convenience	Individuals earning a lower income or experiencing poverty may have been unable to support costs associated with vaccination, like transport or time off to recover [32]
Disability	Complacency	Individuals with a disability may have been less complacent, as they were at greater risk of a severe outcome of COVID-19
	Convenience	Individuals with a disability may have had greater difficulty in accessing a vaccine (e.g., inadequate transport, services lacking disability accommodations). Additionally, hesitancy may vary depending on disability type [40]
Age	Complacency	Younger individuals were at a lower risk of severe outcomes and thus would be more complacent about the vaccination [41, 42]
Essential worker	Complacency	Essential workers may have been at higher risk of exposure to COVID-19 and thus chosen to receive the vaccine more quickly; however, there may be great variation in hesitancy based on the profession of the essential worker (i.e., healthcare versus non-healthcare) [43]
Had physical contact with high-risk person	Complacency	Those who had frequent contact with high-risk individuals may have been more likely to receive a vaccine, to minimize their risk of infecting a high-risk individual [44]
High-risk medical condition	Complacency	Individuals with a high-risk medical condition may have been less complacent, as they were at greater risk of a severe outcome of COVID-19. However, individuals more likely to be hesitant for other reasons (e.g., race, income level) may be more likely to have a high-risk condition and thus remain hesitant [45]
Increased anxiety	Complacency	Individuals with increased anxiety during the pandemic may have been more likely to receive the vaccine to minimize this anxiety [46]
Increased depression	Complacency	Individuals reported feeling more depressed during the pandemic than prior to may have been experiencing this due to increased social isolation. Hence, receiving a vaccine may have increased their ability to participate in their pre-pandemic, day-to-day activities [47]
Prior COVID-19 symptoms	Complacency	If individuals previously experienced symptoms of COVID-19, they may have been more motivated to receive a vaccine to reduce the future likelihood of such symptoms or they may consider themselves as being less susceptible to the effects of COVID-19 [48]
Prior COVID-19 diagnosis	Complacency	If individuals previously experienced COVID-19, they may have been more motivated to receive a vaccine to reduce of contracting the disease again or may consider themselves less susceptible to the effects of COVID-19 [48]
Spent more time in crowds	Complacency	Individuals who were willing to spend time in crowds may have been less concerned about contracting COVID-19 and thus less motivated to get the vaccine [49]
Spent more time with family	Complacency	Individuals who spent more time at home with family during the pandemic may have experienced disruptions to their normal social routines during the pandemic may have seen the vaccine as a means to regain their prior social experiences [50]
Unable to visit sick/elderly relatives	Complacency	Individuals may have thought of the vaccine as a means to be able to visit their sick/elderly relatives once again (i.e., “get back to normal”) [50]
Attended indoor religious services	Complacency	Individuals attending indoor gatherings may have been less concerned about contracting COVID-19 and thus less motivated to get the vaccine
	Confidence	Affiliation with certain religious groups was associated with lower trust in COVID-19 vaccines due to mis- and disinformation [51]
Race/ethnicity	Confidence	Historic mistrust of medical interventions, including vaccines, exists disproportionately among people of color, particularly Black and indigenous individuals [10, 32]
Sex assigned at birth	Confidence	Females of reproductive age have been demonstrated to have lower confidence in vaccines; however, vaccine rates overall in the US are higher for females and seek out healthcare more frequently than men [41, 52–54]

Unweighted data were analyzed, and complete case analysis (where only data from those without missing values were used) was undertaken due to low percent of missing data. Descriptive analyses were tabulated by site. Chi-square tests were used to assess significant associations between categorical variables and *t*-tests and Wilcoxon rank sum tests for continuous ones. All factors significantly associated with vaccine hesitancy in bivariate analyses were considered in multivariable logistic regression modeling. A backward stepwise procedure was used for variable selection, with *p* < = 0.05 used as the threshold for retention in the model. The final model was adjusted for site to account for any differences by site that may not be captured by other demographic variables. This study used de-identified, secondary data and thus was deemed exempt from IRB oversight.

## Results

Between January 19, 2021, and July 31, 2021, there were 11,559 adults aged 18 years and older who were enrolled at community venues in the nine sites included in this analysis, of whom complete data on vaccine hesitancy were available for 11,429 (99%). Among these individuals, median age by site ranged from 46 to 54 years old (Table 2). In seven of the nine sites, the majority of participants were unemployed (range across sites, 39.5–77.7%). Household income was low among participants, with the highest proportion (23.5–53.0%) reporting earning less than $25,000 per year at every site. Most participants (54.5–75.7%) reported a medical condition that would put them at elevated risk for severe COVID-19 outcomes. The prevalence of vaccine hesitancy at each of the nine sites ranged from 8.7 to 31.1%.

**Table 2:** Demographic characteristics of adults, age 18 y + , enrolled in COMPASS, 2021, by study site (*n* (%)) unless otherwise specified

	Baltimore (*n* = 853)	Bronx (*n* = 2079)	Chicago (*n* = 1224)	Harlem (*n* = 2254)	Houston (*n* = 1122)	New Orleans (*n* = 1600)	NY-Columbia (*n* = 506)	Newark (*n* = 828)	Pittsburgh (*n* = 1093)	Total (*n* = 11,559)
Age, years (median (IQR)) (missing = 0)	54 (43–60)	51 (36–61)	50 (35–57)	52 (35–61)	46 (35–57)	49 (33–60)	48 (32–59)	53 (41–60)	51 (33–64)	51 (36–50)
Sex (missing = 6)										
Male	441 (51.7)	1017 (48.9)	788 (64.4)	1124 (49.9)	434 (38.7)	832 (52.0)	205 (40.5)	440 (53.1)	518 (47.4)	5799 (50.2)
Female	412 (48.3)	1062 (51.1)	436 (35.6)	1130 (50.1)	688 (61.3)	766 (47.9)	301 (59.5)	288 (46.9)	571 (52.2)	5734 (49.8)
Race (missing = 0)										
White	44 (5.2)	68 (3.3)	155 (12.7)	123 (5.5)	342 (20.9)	443 (27.7)	89 (17.6)	47 (5.7)	652 (59.6)	1855 (16.0)
Black	759 (89.0)	748 (36.0)	554 (45.3)	1,349 (59.9)	424 (37.8)	985 (61.6)	95 (18.8)	651 (78.6)	244 (22.3)	5809 (50.3)
Asian	2 (0.2)	9 (0.4)	16 (1.3)	44 (1.9)	42 (3.7)	12 (0.7)	42 (8.3)	7 (0.8)	52 (4.8)	226 (2.0)
White Hispanic	4 (0.5)	166 (8.0)	80 (6.5)	42 (1.9)	380 (33.0)	36 (2.3)	47 (9.3)	19 (2.3)	40 (3.7)	804 (7.0)
Other/multiple	44 (5.2)	1088 (52.3)	419 (34.2)	696 (30.9)	52 (4.6)	124 (7.7)	233 (46.05)	104 (12.6)	105 (9.61)	2965 (24.8)
Employment (missing = 388)										
Employed full time	117 (14.7)	333 (16.2)	271 (22.5)	445 (20.2)	337 (32.5)	477 (31.0)	188 (40.5)	181 (22.3)	451 (43.7)	2811 (25.2)
Employed part time	60 (7.5)	253 (12.3)	183 (15.2)	238 (10.8)	83 (8.0)	176 (11.4)	176 (11.4)	64 (7.9)	173 (16.8)	1310 (11.7)
Not employed	618 (77.7)	1473 (71.5)	751 (62.3)	1518 (69.0)	618 (59.5)	887 (57.6)	212 (43.2)	565 (69.7)	408 (39.5)	7.050 (63.1)
Housing (missing = 292)										
Lives in a house/apartment	776 (93.6)	1937 (94.6)	1132 (96.2)	2025 (91.7)	821 (75.9)	1444 (93.5)	488 (97.4)	720 (89.0)	1047 (98.1)	10,390 (92.2)
Unstable housing situation	53 (6.4)	111 (5.4)	45 (3.8)	184 (8.3)	261 (24.1)	101 (6.5)	13 (2.6)	89 (11.0)	20 (1.9)	877 (7.8)
Is an essential worker** (missing = 40***)	107 (63.3)	446 (76.5)	236 (52.2)	489 (71.9)	213 (51.6)	314 (48.6)	197 (70.9)	166 (69.2)	377 (60.8)	2545 (62.4)
Household income (missing = 0)										
< $25,000	452 (53.0)	762 (36.6)	567 (46.3)	1026 (45.5)	512 (45.6)	577 (36.1)	167 (33.1)	289 (34.9)	257 (23.5)	4609 (39.9)
$25,000–$49,999	79 (9.3)	211 (10.1)	174 (14.2)	353 (15.7)	198 (17.6)	217 (13.6)	85 (16.8)	84 (10.1)	159 (14.5)	1560 (13.5)
$50,000–$99,999	29 (3.4)	107 (5.1)	95 (7.8)	192 (8.5)	107 (9.5)	189 (11.8)	107 (21.1)	56 (6.8)	206 (18.8)	1088 (9.4)
> = $100,000	8 (0.9)	5 (0.2)	26 (2.1)	40 (1.8)	44 (3.9)	51 (3.2)	32 (6.3)	8 (1.0)	108 (9.9)	322 (2.9)
Did not report	285 (33.4)	994 (47.8)	362 (29.6)	643 (28.5)	261 (23.3)	566 (35.4)	115 (22.7)	391 (47.2)	363 (33.2)	3980 (34.4)
Reported high-risk medical condition* (missing = 0)	646 (75.7)	1306 (62.8)	754 (61.6)	1313 (58.2)	630 (56.1)	1009 (63.1)	276 (54.5)	593 (71.6)	683 (62.5)	7210 (62.4)
Reported disability (missing = 0)	314 (36.8)	574 (27.6)	260 (21.2)	501 (22.2)	190 (16.9)	300 (18.7)	104 (20.5)	212 (25.6)	191 (17.5)	2646 (22.9)
Vaccine hesitancy (missing = 130)										
Non-hesitant	594 (70.8)	1474 (71.3)	962 (79.6)	1541 (68.9)	910 (81.4)	1233 (77.8)	460 (91.3)	612 (74.2)	910 (87.1)	8696 (76.1)
Vaccine hesitant	245 (29.2)	594 (28.7)	247 (20.4)	695 (31.1)	208 (18.6)	352 (22.2)	44 (8.7)	213 (25.8)	135 (12.9)	2733 (23.9)

*Including asthma, other chronic lung disease, heart disease, hypertension (high blood pressure), cancer chemotherapy in the last 12 months, immunosuppression condition, HIV, diabetes, kidney or renal disease, liver disease, sickle cell disease, obesity, mental health condition, substance use disorder, other chronic medical condition

**Percent represents percent of those employed

***Missing value represents number missing of those reporting employment

In bivariate analyses, younger age, being Black (Hispanic or non-Hispanic), not being employed, unstable housing, lower income, reporting a physical, mental, and/or emotional disability, spending time in crowds, and taking care of someone in the home were each positively associated with vaccine hesitancy (Supplementary Table 1). By contrast, being Asian (compared to White), having experienced COVID-19 symptoms, previously testing positive for COVID-19, reporting a high-risk medical condition, reporting increased anxiety, reporting increased depression, having spent more time with family during the pandemic, and not being able to visit sick or loved ones because of the pandemic were inversely associated with vaccine hesitancy. Sex assigned at birth, being an essential worker, having contact with someone who was high risk, and attending indoor religious services were not associated with vaccine hesitancy.

In the final model (Table 3), after adjusting for enrollment site, being Black (compared to White) (odds ratio (OR), 1.82 95% confidence interval (C.I.) [1.53, 2.16], *p* < 0.001), younger age (OR, 1.03 per year younger [95% C.I. 1.03, 1.04], *p* < 0.001), being unemployed (OR, 1.23 [95% C.I. 1.08, 1.40], *p* = 0.002), reporting a disability (OR, 1.19 [95% C.I. 1.05, 1.33], *p* = 0.004), and caring for someone at home because of the pandemic (OR, 1.36 [95% C.I. 1.22, 1.51], *p* < 0.001) were associated with increased odds of vaccine hesitancy. Asian race compared to White race (OR, 0.38 [95% C.I. 0.22, 0.67], *p* = 0.001) and being in a higher income category (ORs, 0.75 [95% C.I. 0.61, 0.94] for $25,000–$49,999 compared to < $25,000, *p* = 0.011 to 0.45 [95% C.I. 0.28, 0.70] for > = $100,000 compared to < 25,000, *p* < 0.001) were associated with a lack of vaccine hesitancy. Additionally, reporting increased anxiety during the pandemic (OR, 0.76 [95% C.I. 0.69, 0.84], *p* < 0.001), having spent more time with family during the pandemic (OR, 0.82 [95% C.I. 0.74, 0.91], *p* < 0.001), and being unable to visit a sick or older relative (OR, 0.75 [95% C.I. 0.68, 0.83], *p* < 0.001) were also associated with a lack of vaccine hesitancy. Though nearly 30% of individuals did not report their income, missing data on income was not associated with hesitancy.

**Table 3:** Fully adjusted multivariable logistic model for COVID-19 vaccine hesitancy (*n* = 10,633)*

Factor	Odds ratio (95% confidence interval)	*p* value
Age (per year younger)	1.03 (1.03, 1.04)	** < 0.001**
Race		
White, non-Hispanic	Ref	
Black	1.82 (1.53, 2.16)	** < 0.001**
Asian	0.38 (0.22, 0.67)	**0.001**
Hispanic White	1.08 (0.84, 1.40)	0.535
Other/multiple	1.19 (0.98, 1.44)	0.071
Employment status		
Full time employment	Ref	
Part time employment	1.16 (0.97. 1.37)	0.101
No employment	1.23 (1.08. 1.40)	**0.002**
Housing status		
House or apartment	Ref	
Unstable housing	1.60 (1.36, 1.89)	** < 0.001**
Income		
< $25,000	Ref	
$25,000–$49,999	0.75 (0.61, 0.94)	**0.011**
$50,000–$99,999	0.69 (0.56, 0.84)	** < 0.001**
> = $100,000	0.44 (0.28, 0.70)	** < 0.001**
Did not report	0.98 (0.88. 1.08)	0.664
Reports a disability		
Yes	1.19 (1.05, 1.33)	**0.004**
No	Ref	
Reports increased anxiety		
Yes	0.76 (0.69, 0.84)	** < 0.001**
No	Ref	
Care for someone at home		
Yes	1.36 (1.22, 1.51)	** < 0.001**
No	Ref	
Spent more time with family		
Yes	0.82 (0.74, 0.91)	** < 0.001**
No	Ref	
Could not visit sick or elderly relatives		
Yes	0.75 (0.68, 0.83)	** < 0.001**
No	Ref	

*Adjusting for site and all other variables in the model

Values in bold indicate statistical significance at *p *<0.05

## Discussion

In this sub-study of COMPASS, a population-based cross-sectional COVID-19 serosurvey of individuals recruited from community venues during the first half of 2021, COVID-19 vaccine hesitancy varied across nine largely urban sites in the US from 8.7 to 31.1%. This may be due to a combination of demographic variation, as well as policy-level differences in terms of interventions implemented, approaches to COVID-19 mitigation, and vaccine sentiment generally. Moreover, what was known about vaccines and their accessibility changed significantly throughout the course of this study, as did mandates and other vaccine directives; hence, these findings likely reflect early in the pandemic and thus are particularly illustrative of vaccine rollout early in an emergency setting. Interestingly, the sites with the lowest hesitancy (New York-Columbia) and highest hesitancy (Harlem) were geographically proximal. However, these sites may have yielded different population samples, due to the Columbia site recruiting largely closer to the medical center; thus, the demographics of the two populations recruited differed.

This unique community cohort included demographic groups often underrepresented in studies of vaccine hesitancy, and we found several demographic factors and COVID-19-related behaviors were associated with a 62% decrease to an 82% increased odds of COVID-19 vaccine hesitancy at a time when vaccine rollout was occurring among the general population. Certain factors, like younger age and being Black, have been previously identified in the literature as being associated with vaccine hesitancy [8, 55]. However, we identified additional sociodemographic factors associated with vaccine hesitancy, including not being employed, having lower income, and housing instability.

When considering vaccine hesitancy in the framework of complacency, confidence, and convenience, our findings suggest that convenience may have played a major role during this early stage of vaccine availability, since those facing social or economic marginalization were more likely to be vaccine hesitant. Individuals with lower income and unstable housing may have faced structural difficulties in obtaining vaccines, which indicates an issue of convenience. The study design of COMPASS highlighted the significance of these additional sociodemographic characteristics in understanding vaccine hesitancy. The survey question asked how likely individuals were to receive an approved COVID-19 vaccine in the future; hence, the findings suggest that those who were marginalized due to their income or other sociodemographic factors may not have perceived the vaccines as accessible. Though there were numerous public health interventions aimed at reducing barriers to vaccine access (e.g., subsidizing transport to vaccine clinics, pop-up vaccine clinics, not requiring insurance or payment for the vaccine), our findings indicate these interventions may not have been adequate in addressing vaccine hesitancy among those facing multidimensional forms of marginalization.

A novel finding was that having a disability was associated with increased vaccine hesitancy—a finding opposite to what was hypothesized. For those with disabilities, this may indicate concerns about access (i.e., inadequate disability accommodations). Additionally, though the CDC recommended vaccines for people with disabilities, it is possible that individuals may have been concerned about the novel mRNA technology impacting pre-existing conditions. Additionally, those taking care of someone at home were found to have higher vaccine hesitancy—another finding that was not aligned with our hypothesis. This may have been because those taking care of someone at home were unable to find alternative care for those individuals and thus did not feel they would be able to be vaccinated. Alternatively, they may have been concerned about side effects impairing their ability to care for their loved ones. However, the questionnaire did not ask participants to specify the person for whom they were providing care; thus, it is not possible to draw definitive conclusions. Unpacking vaccine decisions among these populations, and thus determining if hesitancy was due to convenience or confidence, will be important to understanding how to improve vaccination rates more quickly.

Though the present findings add to the importance of convenience as a driving factor of hesitancy, the association between decreasing age and increasing hesitancy suggests that younger people likely had a lower perceived risk of illness and demonstrates the importance of complacency in vaccine hesitancy. Furthermore, those who reported increased anxiety, and who spent more time with family at home, or who were unable to visit a sick and elderly relative were less likely to report vaccine hesitancy. People with anxiety might be more likely to get the vaccine because it would lessen their anxiety. Moreover, changes to family time or visitation may be another reason to get the vaccine to allow for a return to previous dynamics. Taken together, this further supports the importance of the pillar of complacency, where individuals who could not return to pre-pandemic activities without being vaccinated were more likely to engage in vaccination.

The role of confidence was also highlighted by our findings. Black people had nearly two times the odds of hesitancy than their White counterparts, suggesting the importance of confidence in lessening vaccine hesitancy among populations who have experienced historical medical injustices. Conversely, Asian people had lower odds of vaccine hesitancy compared to White people, possibly suggesting greater trust among this group or more cohesive outreach and messaging, after accounting for socioeconomic factors. Though the impacts of race on vaccine hesitancy have been previously demonstrated, including in our parent study [10 32 33], it is important to note that this association remained significant even after accounting for social and structural factors that would limit access to the vaccine and that would likely be associated with race through some pathways of social determinants of health. However, the survey did not ask about experiences of healthcare discrimination or adverse healthcare experiences, which may be leading to hesitancy. Hence, healthcare providers and public health practitioners should be aware of multiple components of vaccine hesitancy among people experiencing social/structural marginalization and who are members of racial and ethnic minority groups.

Interestingly, certain variables showed no association with vaccine hesitancy. Sex has previously been associated with vaccine hesitancy, with females of reproductive age expressing more hesitancy due to concerns about the potential impact of the vaccine on fertility [52, 53]. However, in general, more females than males are vaccinated across the US [41]. We did not examine the interaction between age and sex and their relationship with hesitancy, though future research may seek to unpack whether females of reproductive age have different vaccine attitudes than their older and younger counterparts. Moreover, our findings indicate that increased exposure (being an essential worker or having contact with individuals who were high risk) did not impact vaccine hesitancy, which is surprising as it is expected that individuals in these groups would be more concerned about contracting COVID-19. This may indicate the inadequacy of the messaging on the importance of vaccination for individuals in these high-risk categories. Finally, while religiosity has previously been shown to be associated with lower vaccine receipt and higher hesitancy [51], in this analysis, attending religious services indoors was not associated with increased hesitancy. This may be due to religious services still being restricted in some areas early in vaccine rollout. Hence, this finding may be driven more by restrictions on services rather than by the association between religiosity and vaccine hesitancy.

The findings from this study are subject to several limitations. Though time-location sampling was utilized, we did not adjust for sampling weights in this analysis, as we wanted to fully capture those facing social and economic vulnerabilities; as such, this sample may not meet the probabilistic assumptions of a typical time-location sample. As with the parent study [33], the results do not reflect the entire US population but instead reflect the individuals who were well enough to attend commonly frequented venues in nine diverse, largely urban communities surrounding the participating clinical research sites. Though we may have captured information from participants in rural and peri-urban geographies who spent time in urban areas, we were unable to distinguish between trends in different geographies beyond recruitment site alone. Participants were also not asked to specify why they were more or less likely to receive an approved COVID-19 vaccine, limiting our ability to understand vaccine attitudes. Moreover, though questions were asked related to vaccine hesitancy, this was not the primary objective of the study. Thus, some known predictors of vaccine hesitancy (such as political affiliation) were not captured. Finally, missing data methods were not applied to the sample data and thus there may be resulting biases if missingness is not completely at random. Certain variables, such as income, were missing for over 30% of participants. However, in these instances, missingness was not associated with the outcome of hesitancy.

A major strength of this study was its enrollment of individuals who are not typically engaged in COVID-19 research, including individuals earning a lower income, those with unstable housing, and people of color [32, 37]. Additionally, the wide geographic distribution allows extrapolation to similar populations across different regions of the US. Even after controlling for recruitment site, many commonalities remained in terms of predictors of vaccine hesitancy, indicating the importance of engaging diverse populations in vaccine outreach initiatives broadly.

Moreover, we conducted several sensitivity analyses to ensure model robustness. A forward selection procedure with entry at *p* < 0.05 was used to determine if the same variables were selected into the final model. Additionally, the same backwards selection technique was applied to a random sample of 70% of the data to determine if the same variables were retained. This was iterated 50 times and the counts of the models produced were tallied. In the forward selection sensitivity analysis, the variables were retained in the final model. When performing 50 iterations of a backwards selection procedure on a random 70% of the sample, age, race, housing status, income, anxiety, and caring for someone at home were all retained in every model and 17 (34%) of the models were the same as the original model. Though disability status was excluded in 22 (44%) of the models, this was likely due to only 23% of people reporting a disability; thus, a 70% subset will likely produce variable fluctuation in the final model from a model selection process. We also used multiple steps in our final model selection, including the use of a theoretical model, performing bivariate analyses, and our backwards selection process. Thus, we are highly confident in the model.

Our findings highlight the importance of convenience as a pillar of vaccine hesitancy, particularly among individuals facing marginalization and in the context of a health emergency. While there were many programs to improve convenience (e.g., no cost vaccines, subsidized or free transport to vaccine sites, pop-up vaccine sites), barriers to vaccination clearly remained. Future research should examine the awareness, utilization, and effectiveness of policies surrounding vaccine convenience.

In conclusion, among underrepresented populations, socioeconomic marginalization was associated with vaccine hesitancy during the early phases of COVID-19 vaccine rollout. These findings suggest that lack of convenience was a driver of vaccine hesitancy and point toward the importance of specific interventions improving vaccine convenience in pandemic and other emergency situations.

## Supplementary Information

Below is the link to the electronic supplementary material.

Supplementary file1 (DOCX 51 KB)

## Data Availability

Data is available upon reasonable request to HPTN-Data-Access@scharp.org.

## Abbreviations

COMPASS: Community Prevalence of SARS-CoV-2 study
OR: Odds ratio
CI: Confidence interval
