# Supplementary material for: Vaccine Hesitancy at Nine Community Sites Across the United States, Early in COVID-19 Vaccine Rollout
Source: J Racial Ethn Health Disparities. 2024 Sep 12;12(6):3721–32. doi: 10.1007/s40615-024-02172-0 (PMC12644162; doi:10.1007/s40615-024-02172-0)
Supplement: Supplementary file 1 — Supplementary file1 (DOCX 51 KB) [file 40615_2024_2172_MOESM1_ESM.docx]

Supplementary Table 1: Bivariate analysis of factors associated with COVID-19 vaccine hesitancy

| Factor | Non-Hesitant (N=8,696), n(%) | Hesitant (N=2,733) n(%) | p-value |
| --- | --- | --- | --- |
| Site (missing=0)  Baltimore  Bronx  Chicago  Harlem  Houston  New Orleans  New York- Columbia  Newark  Pittsburgh | 594 (6.8)  1,474 (16.9)  962 (11.1)  1,541 (17.7)  910 (10.5)  1,233 (14.2)  460 (5.3)  612 (7.0)  910 (10.5) | 245 (9.0)  592 (21.7)  247 (9.0)  695 (25.4)  208 (7.6)  352 (12.9)  44 (1.6)  213 (7.8)  135 (4.8) | **<0.001^1^** |
| Age (median, IQR) (missing=0) | 52 (37-61) | 44 (33-56) | **<0.001^2^** |
| Sex assigned at birth (missing=6)  Male  Female | 4,327 (49.8)  4,355 (50.2) | 1,408 (51.6)  1,322 (48.4) | 0.101**^1^** |
| Race (missing=0)  White Non-Hispanic  Black  Asian  White Hispanic  Other/ Multiple | 1,548 (17.8)  4,111 (47.3)  209 (2.4)  650 (7.5)  2,178 (25.0) | 272 (9.9)  1,635 (59.8)  15 (0.5)  147 5.4)  664 (24.3) | **<0.001^1^** |
| Employment (missing=388)  Employed Full Time  Employed Part Time  Not Employed | 2,248 (26.7)  964 (11.5)  5,210 (61.8) | 531 (20.2)  326 (12.4)  1,776 (67.4) | **<0.001^1^** |
| Housing (missing=292)  Lives in a House/ Apartment  Unstable Housing Situation | 7,944 (93.3)  573 (6.7) | 2,328 (88.7)  296 (11.3) | **<0.001^1^** |
| Is an essential worker (missing=40) | 1,975 (62.1) | 541 (63.5) | 0.457**^1^** |
| Household Income (missing=0)  <$25,000  $25,000-$49,999  $50,000-$99,999  >=$100,000  Did not report* | 3,369 (38.7)  1,208 (13.9)  911 (10.5)  294 (3.4)  2,914 (33.5) | 1,191 (43.6)  336 (12.3)  163 (6.0)  25 (0.9)  1,018 (37.2) | **<0.001^1^** |
| Reported high risk medical condition** (missing=0 | 5,515 (63.4) | 1,629 (59.6) | **<0.001^1^** |
| Reported disability (missing=0) | 1,945 (22.4) | 675 (24.7) | **0.011^1^** |
| Ever had COVID-19 symptoms since 11/2019 (missing***=3061) | 1,269 (19.4) | 319 (17.0) | **0.021^1^** |
| Ever had positive COVID-19 diagnosis (missing=83) | 794 (9.2) | 214 (7.9) | **0.037^1^** |
| Spent time in crowds (mean (SD), scale of 1-5****) (missing=155) | 2.3 (1.3) | 2.4 (1.4) | **0.012^3^** |
| Had physical contact with someone high risk (mean (SD), scale of 1-5) (missing=106) | 1.9 (1.5) | 2.0 (1.6) | 0.098^3^ |
| Attended religious services indoors (mean (SD), scale of 1-5) (missing=171) | 1.6 (1.2) | 1.6 (1.1) | 0.368^3^ |
| Reports more anxiety than usual (missing=135) | 5,300 (61.3) | 1,463 (54.0) | **<0.001^1^** |
| Reports more depression than usual (missing=159) | 4,090 (47.4) | 1,207 (44.6) | **0.011^1^** |
| Cared for someone in their home (missing=105) | 2,149 (24.8) | 861 (31.6) | **<0.001^1^** |
| Spent more time with family (missing=93) | 4,797 (55.3) | 1,460 (55.6) | **0.013^1^** |
| Was unable to visit sick or elderly relatives (missing=107) | 4,364 (44.7) | 1,209 (51.0) | **<0.001^1^** |

Note: “missing” represents the number of participants with missing information on the variable of interest

^1^ Chi Square test

^2^ Wilcoxon rank sum test

^3^ T-test

* Those who did not know or did not wish to report their income were categorized separately, due to the high level of non-response for this question

**Including: asthma, other chronic lung disease, heart disease, hypertension (high blood pressure), cancer chemotherapy in the last 12 months, immunosuppression condition, HIV, diabetes, kidney or renal disease, liver disease, sickle cell disease, obesity, mental health condition, substance use disorder, other chronic medical condition

*** Question added on midway through study

****Where 1=never and 5=all the time
